# Tools and scores for general and cardiovascular perioperative risk assessment: a narrative review

**DOI:** 10.1590/0100-6991e-20223124

**Published:** 2022-03-11

**Authors:** CAIO MAZZONETTO TEÓFILO DE MORAES, LUISA DE MENDONÇA CORRÊA, RICARDO JAYME PROCÓPIO, GABRIEL ASSIS LOPES DO CARMO, TULIO PINHO NAVARRO

**Affiliations:** 1 - Universidade Federal de Minas Gerais - Belo Horizonte - MG - Brasil; 2 - Faculdade Ciências Médicas de Minas Gerais - Belo Horizonte - MG - Brasil; 3 - Universidade Federal de Minas Gerais, Hospital das Clínicas, Unidade Endovascular - Belo Horizonte - MG - Brasil; 4 - Universidade Federal de Minas Gerais, Departamento de Clínica Médica - Belo Horizonte - MG - Brasil; 5 - Universidade Federal de Minas Gerais, Departamento de Cirurgia - Belo Horizonte - MG - Brasil

**Keywords:** Perioperative Period, Risk Assessment, Postoperative Complications, Decision Support Techniques, General Surgery, Sistemas de Apoio a Decisões Clínicas, Período Perioperatório, Complicações Intraoperatórias, Complicações Pós-Operatórias, Cirurgia Geral

## Abstract

The number of surgical procedures in the world is large and in Brazil it has been expressing a growth trend higher than the population growth. In this context, perioperative risk assessment safeguards the optimization of the outcomes sought by the procedures. For this evaluation, anamnesis and physical examination constitute an irreplaceable initial stage which may or may not be followed by complementary exams, interventions for clinical stabilization and application of risk estimation tools. The use of these tools can be very useful in order to obtain objective data for decision making by weighing surgical risk and benefit. Global and cardiovascular risk assessments are of greatest interest in the preoperative period, however information about their methods is scattered in the literature. Some tools such as the American Society of Anesthesiologists Physical Status (ASA PS) and the Revised Cardiac Risk Index (RCRI) are more widely known, while others are less known but can provide valuable information. Here, the main indices, scores and calculators that address general and cardiovascular perioperative risk were detailed.

## RESUMO

O número de procedimentos cirúrgicos no mundo é amplo e no Brasil vem expressando tendência de crescimento superior ao crescimento populacional. Nesse contexto, a avaliação de risco perioperatório resguarda a otimização dos desfechos buscados pelos procedimentos. Para a realização dessa avaliação, a anamnese e exame físico constituem etapa inicial insubstituível, a qual pode ou não ser seguida de exames complementares, intervenções para estabilização clínica e aplicação de ferramentas de estimativa de risco. A utilização destas ferramentas pode ser bastante útil a fim de se obter um dado objetivo para a tomada de decisão pesando-se risco e benefício cirúrgico. As avaliações de risco global e cardiovascular são as de maior interesse no pré-operatório, entretanto informações sobre seus métodos encontram-se dispersas na literatura. Algumas ferramentas como o American Society of Anesthesiologists Physical Status (ASA PS) e Índice de Risco Cardíaco Revisado (RCRI) são mais amplamente conhecidos, enquanto outros são menos conhecidos em nosso meio mas podem fornecer informações valiosas. Aqui detalhou-se os principais índices, escores e calculadoras que abordam risco perioperatório geral e cardiovascular.

## INTRODUCTION

The volume of operations in the world is vast^1^ and Brazil has shown a growing trend in the number of surgical procedures, which is proportionally higher than the population growth^2^. Despite this, the estimate of the need for operations^3^ considerably exceeds the numbers contained in the public records, showing room for expansion.

In this context, perioperative risk assessment is necessary to mitigate the potential impacts of morbidity and health expenses arising from the growing number of surgical procedures and their complications. The risk assessment performed in the preoperative period aims to optimize outcomes from the perioperative period to the patient’s full recovery in the late postoperative period.

The bases are the anamnesis and the physical examination, which are essential and irreplaceable steps to identify comorbidities, indicate additional tests, recommend clinical stabilization and possible contraindications to the operation. After this step, calculators and scores provide physicians and patients with increased objectivity of the risk-benefit assessment before joint decision-making, especially in elective procedures.

Despite the usefulness calculators and scores, they appear dispersed in the literature so that the gathering and detailing of their functioning add didactic and informative value to professionals who will use them, in addition to enabling an analytical view that allows the choice of the best tool for the patient’s preoperative health status.

 The systems approach facilitates the organization of the preoperative risk assessment. This literature review lists and discusses indices, scores, and calculators related to general perioperative and cardiovascular risk that receive greater focus in medical practice. We searched the electronic databases Pubmed/MEDLINE and EMBASE for manuscripts in English and Portuguese. The scope of this review does not include cardiac operations, which have specific risk assessment scores.

### General risk assessment in non-cardiac surgeries

The incidence of complications resulting from non-cardiac procedures is on average between 7% and 11%, reaching 21.4% depending on the location and on the safety measures adopted^4^. The average 30-day mortality rate is between 0.8% and 1.8%^4^ ^,^ ^5^. The ASA PS score and the ACS NSQIP calculator described below are tools capable of predicting the risk of complications and mortality in general, without guidance by organ system.

### American Society of Anesthesiologists Physical Status (ASA PS)

 The American Society of Anesthesiologists (ASA) classification was created in 1941 with the aim of simply determining the clinical status of surgical patients6. The tool was revised in 1963 and became widely used in the preoperative period, due to its simplicity and reproducibility. The patient’s clinical status is assigned a scale between I and VI (Table 1).

**Table 1:** American Society of Anesthesiologists (ASA) Classification 10. Complications and mortality according to Hackett et. al.^9^.

Class	Description	Complications (%)	Mortality (%)
I	Healthy	2	0.02
II	Mild systemic disease	5	0.14
III	Severe systemic disease	14	1.41
IV	Severe systemic disease with constant threat to life	37	11.14
V	Dying, with no expectation of survival without the operation	71	50.87
VI	Declared brain death, awaiting removal of organs for donation		
E	Addition of the letter "E" denotes surgical emergency		

There are criticisms of the use of the ASA PS as a surgical risk assessment, since it was not created with the aim of assigning risk and there may be interprofessional variation in the patients’ classification^7^. However, the tool is simple, fast, easy to use, independent of complementary tests, can be a good predictor of risk of death in conditions of low mortality^8^, and is an independent predictor of postoperative complications and mortality^9^.

### American College of Surgeons National Surgical Quality Improvement Program Risk Calculator (ACS NSQIP)

This calculator was initially developed between 2009 and 2012 in the United States based on data from 393 hospitals and about 1.4 million patients, with the objective of becoming a universal tool for estimating surgical risk^11^. It uses 21 patient variables, including the type of operation intended, and delivers the risk of nine main outcomes within 30 days of the procedure, which are summarized in Table 2. It is currently available online, free of charge, and in English (https://riskcalculator.facs.org/RiskCalculator/), where one can drill down to each calculator item. Although there may be risk variation between physicians^12^, the calculator allows for a small adjustment. This tool has already been analyzed in the context of different types of operations and the results regarding the ability to predict outcomes are variable, in general with good prediction of serious outcomes such as death, renal failure, and cardiac complications, but with poor performance for other outcomes^13^ ^-^ ^15^.

**Table 2:** ACS NSQIP calculator variables and outcomes. The type of operation is added to these variables to calculate the risk.

Variables		Outcomes
Age	Diabetes	Death
Sex	Hypertension	Any Complication
Performance Status	Cardiac Insufficiency	Pneumonia
Emergency (yes/no)	Dyspnea	Cardiac Complication
ASA class	Smoking	Surgical Site Infection
Chronic Steroid Use	COPD	Urinary Tract Infection
Ascites	Dialysis	Venous Thromboembolism
Sepsis	Acute Kidney Failure	Renal Failure
Ventilator-dependence	Weight	Serious Complication
Disseminated Cancer	Height	

ACS NSQIP: American College of Surgeons National Surgical Quality Improvement Program; ASA: American Society of Anesthesiologists; COPD: Chronic Obstructive Pulmonary Disease.

### Cardiovascular risk for non-cardiac operation

Myocardial lesions occur in 13%5 of non-cardiac surgeries and increases the risk of complications such as heart failure, stroke, and cardiac arrest, accounting for 34% of perioperative deaths^16^. Furthermore, cardiac complications determine a prolonged length of stay after the surgical procedure^17^. For these reasons, cardiovascular assessment has the largest number of validated algorithms and scores to date.

### Cardiac Risk Index - Goldman index

The Cardiac Risk Index (CRI) was described in 1977 as the first multifactorial model specific for perioperative cardiac complications in non-cardiac procedures. This model categorizes the patient into four classes (I to IV) based on predefined scores for clinical, electrocardiographic, and laboratory factors, as well as type of operation (Tables 3 and 4). Outcomes considered are myocardial infarction, pulmonary edema, ventricular tachycardia within six days after surgery, and death from cardiac causes^18^.

**Table 3:** CRI criteria and scores^18^.

Criteria		Points
1	Age >70 years	5
2	Myocardial infarction in the previous 6 months	10
3	Presence of S3 or jugular stasis	11
4	Severe aortic stenosis	3
5	Non-sinusal rhythm or premature atrial contraction on the last ECG	7
6	>5 ventricular extrasystoles per minute at any time prior to surgery	7
7	PaO2 <60 or PaCO2 >50mmHg; K+ <3mEq/L or HCO3- <20mEq/L; urea >107.5mg/dL or creatinine >3mg/dL; Abnormal AST, signs of chronic liver disease or bedridden patient due to non-cardiac cause	3
8	Intraperitoneal, intrathoracic, or aortic operation	3
9	Emergency operation	4

CRI: Cardiac Risk Index - Goldman Index; ECG: electrocardiogram; S3: third heart sound on cardiac auscultation.

**Table 4:** CRI classes and respective risks of complications and cardiac death^18^.

Class	Punctuation	Complications* (%)	Cardiac mortality (%)
I	0-5	0.7	0.2
II	6-12	5	2
III	13-25	11	2
IV	>26	22	56

*Life-threatening complications (myocardial infarction, pulmonary edema, or ventricular tachycardia, intraoperatively or postoperatively, without progression to cardiac death).

 One of the main limitations of CRI are elective aortic surgeries, in which prediction of complications is underestimated by the score^19^, although its effectiveness as a predictor of long-term mortality in abdominal aortic aneurysm repairs has been reported^20^. In addition, the model has a similar correlation to ASA PS in predicting perioperative mortality, but it is a worse predictor of mortality in low-risk patients, with ASA ≤2^21^

### Detsky Index

 Developed in 1986 as an adaptation for the Goldman’s risk (CRI), it included variables considered clinically important by the authors, in addition to simplifying the scoring scheme, as shown in Table 5. The type of operation was also removed from the index as it was not a patient’s characteristic, and validation included minor procedures, such as cataract extraction or prostate resection^18^. Expected outcomes are myocardial infarction, acute pulmonary edema, tachycardia or ventricular fibrillation requiring electrical cardioversion, death from cardiac causes, and worsening or onset of heart or coronary failure^22^.

**Table 5:** Cardiac risk index adapted by Detsky and colleagues^22^.

Variables	Points
Coronary artery disease	
Myocardial infarction in the last 6 months	10
Myocardial infarction prior to 6 months	5
Angina (Canadian Cardiovascular Society - CCS classification)	
Class III	10
Class IV	20
Unstable angina in the last 6 months	10
Alveolar pulmonary edema	
Past week	10
Anytime	5
Valve disease	
Suspected severe aortic stenosis	20
Arrhythmias	
Non-sinus or sinus rhythm with premature atrial beat on the last preoperative ECG	5
More than 5 ventricular extrasystoles at any time prior to surgery	5
Bad general condition*	5
Age over 70 years old	5
Emergency surgery	10

*PO2 <60 or PCO2 >50mmHg; K <3mEq/L; HCO3 <20mEq/L; urea >107.5mg/dL; Cr >3mg/dL; abnormal AST; signs of chronic liver disease and patient bedridden due to a non-cardiac cause.

The assessment by this method requires knowledge of the pre-test risk of complication of the operation to be performed, which, combined with the Detsky score, determines the posttest risk. The authors propose the use of a nomogram to detail the posttest risk according to the score. In summary, scores below 10 mean that the patient’s risk is less than the pre-test probability of complications from that operation. A score equal to 10 means equal pre- and posttest risk, and greater than 10 expresses that the estimated risk is above the mean^22^ ^,^ ^23^. The Detsky index has already been shown to be equivalent to other perioperative cardiac risk assessment scores but may be inferior to the Revised Cardiac Risk Index (RCRI), described below^24^, in predicting death or stroke, wound complications, and minor neurological complications^25^.

### Revised Cardiac Risk Index (RCRI)

 The index proposed in 1999^24^ was based on the Cardiac Risk Index^18^ and aims at carrying out a simple assessment of the perioperative risk of major cardiac complications in patients aged 50 years and over undergoing major non-cardiac surgeries. Major cardiac complications were defined as acute myocardial infarction, acute pulmonary edema, ventricular fibrillation or primary cardiac arrest, and complete atrioventricular block. The variables independently associated with the increased risk of major cardiac complications were six: high-risk operation, ischemic heart disease, heart failure, history of cerebrovascular disease, insulin-dependent diabetes mellitus, and creatinine >2mg/dL, with an odds ratio between 1.9 and 3.0. High-risk operations were defined as intraperitoneal, intrathoracic, or suprainguinal vascular procedures^24^. For each of the variables, 1 point is attributed, and the classification is made as shown in Table 6. The predictive capacity of this scheme was confirmed in further studies^26^ ^,^ ^27^. It has been one of the most widely used risk assessment scores.

**Table 6:** Variables, classes, and risk of cardiac complications according to RCRI^24^.

Variables (the presence of each adds 1 point)	Class	Risk of complications (%)
High-risk operation	I (0 points)	0.4
Ischemic heart disease	II (1 point)	0.9
Cardiac insufficiency	III (2 points)	7
History of cerebrovascular disease	IV (3 points or more)	11
Diabetes mellitus on insulin		
Serum creatinine >2mg/dL		

RCRI: Revised Cardiac Risk Index

RCRI is well suited for stable patients who will undergo major, non-urgent, noncardiac surgeries, but limited for vascular procedures such as abdominal aortic aneurysm repair^24^ ^,^ ^26^, small surgeries, and very high-risk populations - as in emergency situations^24^. It should be noted that this score predicts cardiac complications and mortality, but it is not a good predictor of overall mortality^26^. One of its limitations is the exclusion of some factors considered clinically important, such as age, functional tolerance, and aortic stenosis^26^. The positive predictive value is greater in younger individuals (that is, under 55 years of age)^28^ but the negative predictive value is high for all ages^29^, that is, class I patients - without any of the six risk factors for index - are well identified by the RCRI as individuals at low risk for cardiac complications.

Despite limitations and the existence of newer tools, RCRI continues to be widely used and is among the perioperative cardiovascular risk assessment indices included in the guidelines of the Brazilian Society of Cardiology (SBC)^30^, American College of Cardiology, American Heart Association (ACC/AHA)^31^, European Society of Cardiology, and European Society of Anesthesiology (ESC/ESA)^32^.

### Fleisher-Eagle

Published in 2001, this assessment resembles the RCRI in the evaluated parameters. However, it does not assign a score, but proposes a flowchart that indicates measures according to the number of risk factors found, to avoid cardiac complications (myocardial infarction, death from cardiac causes). Risk factors considered in the preoperative evaluation are known ischemic heart disease, heart failure, high-risk operation (as in RCRI), diabetes mellitus, renal failure, and inadequate functional status. If all these factors are absent, the authors do not recommend further investigation. With one or two factors present, perioperative use of beta-blocker therapy is recommended for risk reduction, in addition to further investigation of coronary artery disease. With three or more risk factors, the investigation of coronary disease is strongly indicated, and the recommendation for the use of beta-blockers remains. In the case of coronary disease, revascularization prior to the non-cardiac operation is recommended, with percutaneous or open intervention, depending on the affected coronary branches. This proposal was limited because it is a theoretical proposition, without a validation study^33^. 

### Multicenter Perioperative Evaluation Study (EMAPO)

 EMAPO is a Brazilian classification published in 2007 that assesses 27 variables to estimate perioperative risk. Each of these variables is assigned a specific score and the result of the sum of the points of the present variables classifies the patient into one of five risk levels (Tables 7 and 8). On the positive side, the study included validation for the Brazilian population, the inclusion of diseases not addressed by previous risk assessment guidelines, and modern treatment options in its objectives, to determine new variables associated with cardiovascular complications^34^.

**Table 7:** Variables, risk factors, and scores in the EMAPO assessment^34^.

4 points	5 points	10 points	20 points
Patient bedridden or inactive	Age over 70 years old	AMI in the last 6 months but not in the acute phase	Class IV CCS angina
SAH with LVH and ST-T change	AMI for more than 6 months	Transplant operation: kidney or liver recipient	Critical aortic stenosis
Ischemic stroke in the last 3 months	Acute lung edema secondary to heart failure for more than 1 week	Acute lung edema secondary to heart failure for less than 1 week	Class IV heart failure
DM with heart disease, nephropathy, or insulin use	Chronic AF, paroxysmal atrial tachyarrhythmias, or documented non-sustained VT	Sustained supraventricular tachyarrhythmias with increased ventricular response**	AMI in the acute phase
CAD with negative exercise test in the last 3 months	Poor general status*	Angina pectoris currently stable	Recent episode of VF or aborted sudden death in a patient without an automatic implantable defibrillator
Intraperitoneal, intrathoracic, aortic (or its branches), or major orthopedic surgery		Episode of unstable angina pectoris in the last 3 months, currently absent	Transplant operation: lung recipient
Presence of asymptomatic aortic aneurysm		Class III CCS angina	
		Emergency operation	
		Severe mitral stenosis	

SAH: systemic arterial hypertension; LVH: left ventricular hypertrophy; DM: Diabetes mellitus; CAD: coronary artery disease; AMI: acute myocardial infarction; AF: atrial fibrillation; VT: ventricular tachycardia; CCS: Canadian Cardiovascular Society; VF: ventricular fibrillation

*Poor general status: K<3.0mEq/L or HCO2 <20mEq/L, PO2 <60mmHg or PCO2 >50mmHg, urea >50mg/dL or creatinine >2.3mg/dL, high AST, or current liver disease.

**Sustained supraventricular tachyarrhythmias with increased ventricular response: documented repetitive sustained ventricular arrhythmia, history of ventricular fibrillation, episode of sudden death aborted more than 3 months ago, patient with implanted automatic defibrillator.

**Table 8:** Classification of cardiovascular risk according to the EMAPO assessment score^37^.

Risk rating	Punctuation	Estimate of cardiac complications (%)
Very low	0	<1
Low	1-5	<3
Moderate	6-10	<7
High	11-15	7-13
Very high	>15	>13

 The index requires a large amount of information for the application, which can be a limitation^34^. On the other hand, it remains among the indices highlighted by the perioperative cardiovascular assessment guideline of the Brazilian Society of Cardiology, since it was developed and validated for the Brazilian population. The guideline recommends its use in patients without previous severe cardiovascular disease - which must be treated before the operation - and in non-urgent procedures 30.

### National Surgical Quality Improvement Program Myocardial Infarction and Cardiac Arrest (NSQIP MICA)

 NSQIP MICA is a calculator created in 2011 from an extensive database (more than 400,000 patients), multicentric (more than 250 hospitals) and prospective, which aimed to assess risk factors associated with myocardial infarction or cardiac arrest in the peri and postoperative period (up to 30 days after the operation), as this would be a weak point of the risk scores developed so far. These outcomes are considered relevant because, despite being rare (less than 1% in the peri or postoperative period), when they occur, they result in death in 61% of cases within 30 days after the procedure^35^.

 The variables associated with an increased risk of myocardial infarction or cardiac arrest were ASA class, dependent functional status (partially or totally), elevated creatinine (>1.5mg/dL), age, and type of operation. The consideration of dependent functional status in the assessment is a differential of this tool, as it did not appear in other previously published systematized assessments. As this is a more complex calculation, it is used on a website, available at: http://www.surgicalriskcalculator.com/miorcardiacarrest, which can be downloaded or used on the online platform^35^.

Compared to the RCRI, the MICA risk assessment benefits from greater specificity in relation to the procedure performed, but there is no significant association of heart failure with the primary outcomes not covered by the high ASA class and functional dependence^35^, and it remains limited for vascular operations^36^.

 A retrospective observational study found a disagreement between MICA and RCRI assessments in classifying patients at low risk for adverse cardiac events in 30% of cases; the two tools look for different primary outcomes, but disagreement can be problematic, as low-risk patients often undergo surgery without further evaluation^37^. Even so, MICA is among the risk indices recommended by the American (ACC/AHA)^31^ and European (ESC/ESA)^32^ guidelines for perioperative risk assessment.

### Physiological and Operative Severity Score for the enUmeration of Mortality and morbidity (POSSUM)

Published in 1991, POSSUM was developed with 1,372 patients undergoing elective or emergency operations in Liverpool in the years 1988-1989. It is a dual scoring system that combines a 14-item physiological score and a six-item operative severity score, which allows for a more accurate differentiation of risk by type of procedure. The study that originated it showed a good relationship between the predicted risk and the mortality and morbidity outcomes found. However, it was limited to a small population and developed with the aim of assisting in surgical auditing and evaluating quality of care, not validated for the process of decision making^38^. 

The POSSUM assessment has already undergone some adaptations^39^, among which the Portsmouth-POSSUM (P POSSUM)^40^ stands out. It has been observed that the original POSSUM overestimates the prediction of mortality, especially in low-risk patients^41^ ^-^ ^44^, while the P POSSUM is more accurate in predicting postoperative mortality in various surgical scenarios^39^ ^,^ ^41^ ^,^ ^42^ ^,^ ^44^.

### Vascular Study Group of New England Cardiac Risk Index (VSG-CRI)

 The VSG-CRI was proposed in 2010 with the objective of predicting cardiac events specifically for non-emergency vascular operations, seeking efficacy superior to the RCRI in this group, as the latter underestimates the risk of cardiac events in vascular procedures. The proposal is similar in logic to the RCRI, assigning points to a simple score, though the risk factors used are partially different (Tables 9 and 10). The outcomes considered in this evaluation are myocardial infarction, clinically significant arrhythmia, and in-hospital congestive heart failure45. Currently, the VSG-CRI calculator is also available online at http://www.qxmd.com/calculate-online/vascular-surgery, where one can select the specific assessment for each type of vascular procedure. 

**Table 9:** Scores for the VSG-CRI^48^.

Age ≥ 80 years old (+4 points)	CHF (+2 points)	Insulin-dependent diabetes (+1 point)
Age 70-79 years (+3 points)	COPD (+2 points)	Use of β-blockers >1 month (+1 point)
		
age 60-69 years (+2 points)	Cr >1.8mg/dL (+2 points)	Previous surgical or percutaneous coronary revascularization (-1 point)
		
CAD (+1 point)	Current or previous smoking (+1 point)	

CAD: coronary artery disease, defined as a history of myocardial infarction, coronary revascularization, or angina; CHF: congestive heart failure; COPD: chronic obstructive pulmonary disease. Cr: Creatinine level.

**Table 10:** Risk of adverse cardiac outcome according to VSG-CRI^48^.

Points	Risk of adverse cardiac outcome (%)
0-3	2.6
4	3.5
5	6
6	6.6
7	8.9
≥8	14.3

VSG-CRI: Vascular Study Group of New England Cardiac Risk Index.

 The original work proposing the VSG-CRI found greater accuracy than the RCRI in the assessment of risk for vascular operations^45^. Subsequent studies in substantially smaller groups evaluated the VSG-CRI compared to the RCRI in arterial vascular operations and found low accuracy for the RCRI, as expected, but disparity in the results of the VSG-CRI. On the other hand, these studies agree that the VSG-CRI was not adequately accurate in the risk assessment for endovascular repair of abdominal aortic aneurysms (EVAR)^46^ ^-^ ^48^.

## Model for Stroke and Cardiac Risk After Surgery

 The Model for Stroke and Cardiac Risk After Surgery was published in 2021 through a study that included, between derivation and validation groups, 1,165,750 patients from the ACS NSQIP database who underwent surgical procedures between 2007 and 2010. The outcomes predicted by this calculator refer to the first 30 days after surgery and are stroke, major cardiovascular events (myocardial infarction and cardiac arrest), and mortality. The tool requires nine variables: age, history of cerebrovascular disease, history of coronary artery diseas, ASA Class, serum hematocrit, serum sodium, serum creatinine, emergency surgery (yes or no), and type of operation (brain, major vascular, bariatric etc.). According to the original study, performance is excellent and matches or exceeds that of widely used calculators and scores such as the RCRI, MICA, and ACS NSQIP Risk Calculator^49^.

 The main advantage is the inclusion of stroke risk assessment among the outcomes, which is not included in the most used tools. In addition, it includes variables that can be subject to pre-surgical clinical adjustment. It is limited by not considering the presence of atrial fibrillation, an important risk factor for stroke, and by not considering time within the evaluation of the history of cerebrovascular disease. The risk calculation is performed using a computer program, available at http://cvrisk.herokuapp.com/^49^.

## Final remarks

Risk assessment tools have adequate applicability for elective operations, in which patients present clinical stability. Thus, the following algorithm is recommended for risk assessment: in case of urgent operation, apply the appropriate measures for clinical stabilization and risk reduction and proceed with the operation; in case of elective procedure, evaluate the presence of active heart disease (coronary artery disease, heart failure) and, if present, postpone the operation and continue with the care of the disease found until pre-surgical clinical optimization is achieved. In patients without active heart condition who will undergo elective surgery, assess the surgical risk using the scores, considering the respective advantages and limitations as shown in Table 11, and proceed with the operation if the risk is tolerable.

**Table 11:** Advantages and limitations of risk assessment tools.

Tool (year of publication)	Benefits	limitations
ASA PS (1963)^10^	Simple and fast application. Good for identifying low-risk patients	Not developed for risk assessment and performs worse in high-risk situations
ACS NSQIP Calculator (2013)^11^	Evaluates 9 outcomes, assesses 21 variables, almost all clinical	Risk assessment is better for death, renal failure, and cardiac complications than other outcomes
Goldman - CRI (1977)^18^	Simple application	Low performance in aortic operations. There are newer models with similar ratings.
Detsky (1986)^22^	Simple application. Adaptation of the CRI that included more types of operations and outcomes	There are newer models with similar ratings.
RCRI (1999)^24^	Simple application. Latest adaptation of the CRI. Recommended by Brazilian, American, and European risk assessment guidelines	Does not include some clinically important factors (age, functional tolerance, aortic stenosis). Not a good predictor of non-cardiac mortality. Limited performance for vascular operations.
Fleisher-Eagle (2001)^33^	Simple application recommendation algorithm	No validation study
EMAPO (2007)^34^	Validated for the Brazilian population, recommended by the SBC risk assessment guideline	Complex application
MICA Calculator (2011)^35^	Includes functional status in the assessment. Recommended by American and European guidelines	Does not consider preoperative heart failure. Limited performance for vascular operations.
POSSUM (1991)^38^	Developed for application in auditing and quality of care	Complex application. Not validated for clinical application
VSG-CRI (2010)^45^	Developed specifically for vascular operations	Risk assessment for endovascular repair of abdominal aortic aneurysm (EVAR)
Model for Stroke and Cardiac Risk After Surgery (2021)^49^	Assesses outcomes in 30 days. Includes stroke. Uses variables subject to pre-surgical clinical adjustment	Does not consider atrial fibrillation. Does not consider time in the history of cerebrovascular disease.

ASA PS: American Society of Anesthesiologists Physical Status; ACS NSQIP: American College of Surgeons National Quality Improvement Program; CRI: Cardiac Risk Index; RCRI: Revised Cardiac Risk Index; EMAPO: Multicenter Perioperative Evaluation Study; MICA: Myocardial Infarction and Cardiac Arrest; POSSUM: Physiological and Operative Severity Score for the enUmeration of Mortality and morbidity; VSG-CRI: Vascular Study Group of New England Cardiac Risk Index.

 All tools detailed here were developed and should be used for general non-cardiac operations. Some consider the type of operation within the evaluation, which may be of interest to the evaluator, namely ACS Calculator NSQIP, Goldman (CRI), EMAPO, MICA, VSG-CRI (this one specific for vascular operations), and the Model for Stroke and Cardiac Risk After Surgery. It should also be noted that the Goldman, RCRI, and MICA models have limited accuracy for vascular procedures, which is why the VSG-CRI is preferred in the risk assessment of this type of operation. Finally, the combined use of more than one tool can be a strategy adopted by the physician to compose the assessment.

## Study Limitations

 It is noteworthy that the narrative review, the format chosen for aggregating and discussing the information contained herein, is subject to some degree of subjectivity. However, physicians who perform the preoperative assessment will be able to take advantage of this information to adapt the decision-making process about performing a procedure, use calculators and risk scores to complement their assessment, and guide preoperative clinical interventions and the joint decision with the patient.
